# Adsorption of Pb(II) and Cd(II) by Squid *Ommastrephes bartrami* Melanin

**DOI:** 10.1155/2009/901563

**Published:** 2010-01-27

**Authors:** Shiguo Chen, Changhu Xue, Jingfeng Wang, Hui Feng, Yuming Wang, Qin Ma, Dongfeng Wang

**Affiliations:** ^1^College of Food Science and Technology, Ocean University of China, Qingdao, Shandong 266003, China; ^2^Department of Food Science and Technology, Oregon State University, Corvallis, OR 97331-4501, USA

## Abstract

The adsorption of Cd(II) and Pb(II) by squid melanin was investigated. At a metal ion concentration of 2 mM/L, the biosorption efficiency of melanin reached 95% for Cd(II) and Pb(II). The maximum content of bound Cd(II) and Pb(II) was 0.93 mM/g and 0.65 mM/g, respectively. Temperature had no obvious effect on the adsorption of the metals, and in a pH range of 4.0–7.0, the adsorption yield was high and stable. Macrosalts such as NaCl, MgCl_2_, and CaCl_2_ had no obvious effect on the binding of Pb(II) but greatly diminished the adsorption of Cd(II), which indicated that different functional groups in squid melanin are responsible for their adsorption. IR analysis of metal ion-enriched squid melanin demonstrated that the possible functional groups responsible for metal binding were phenolic hydroxyl (OH), carboxyl (COOH), and amine groups (NH). This study reports a new material for the removal of heavy metals from low-strength wastewater.

## 1. Introduction

Several active components, including melanin, a tyrosinase, and an angiotensin-converting enzyme inhibitor, have been identified in squid ink [1–4]. Squid belong to the invertebrate phylum *Mollusca* and rely on the ejection of dark, opaque ink for defense as other cephalopods. The ink consists of a suspension of eumelanin granules in a viscous and colorless medium. Eumelanin is a heterogeneous, generally insoluble polymer developed through enzymatic oxidation of the amino acid tyrosine [5–7]. The production of eumelanin in pigment-generating cells occurs in the specific organelles known as melanosomes. The indolic molecules 5,6-dihydroxyindole and 5,6-dihydroxyinodole-2-carboxylic acid are postulated to be the main monomeric building blocks of eumelanins [5].

Natural eumelanins are reported to have a considerable affinity for metal ions [8–10] and can serve as reservoirs of metal ions (e.g., Ca(II); see [11]) or as traps for heavy metal ions (e.g., Cu(II) and Fe(III); see [12, 13]). It has been suggested that the molecular structure of the pigments could be impaired by high metal concentrations [13]. Such a change could result in the release of heavy metal ions (e.g., Fe(III)) into the cytosol, which could induce cellular damage. Eumelanin's binding capacity, affinity, and sites for metals are important parameters for understanding the nature and consequences of metal-melanin complexation.

Contamination of the environment by heavy metals is of growing concern because of the health risks posed to humans and animals because of exposure to these metals. The vast majority of toxic metal pollutants are waste products of industrial and metallurgical processes. In particular, effluents from electroplating plants, extractive metallurgy processes, and metal-treatment finishing operations contain high concentrations of dissolved metals. In some industrial wastewater, lead ion concentrations can approach 200–250 ppm; by contrast, water-quality standards state that the Pb(II) concentration in wastewater should be reduced to a value of 0.5–1 ppm [14]. The most common methods used to remove metals from wastewater are chemical precipitation, solvent extraction, dialysis or electrodialysis, electrolytic extraction, cementation, reverse osmosis, evaporative methods, ion-exchange resins, carbon adsorption, and dilution [15]. The increasing problem of heavy metal contamination has stimulated a search for new mechanisms to remove these pollutants. Attempts have been made to harness the metal-binding capacity of various microorganisms such as yeast, algae, and bacteria to clean up industrial effluents [16, 17]. 

The melanin in sepia ink has been reported to bind with many ions, including Fe(III), Cu(II), Zn(II), and Ca(II) [18–23]. No reports, however, have discussed the application of squid melanin to remove heavy metals from solution, in particular the biosorption of Cd(II) and Pb(II), which might contribute to the pigment's dark color. We found that after binding with heavy metals squid melanin could form a sediment, leaving the supernatant transparent, which indicates its potential as a new material to remove heavy metals from wastewater. In the present study, we focused on the application of squid melanin to absorb cadmium and lead ions in concocted metal solutions. IR spectrum was applied to reveal the likely binding sites for these two metals in squid melanin.

## 2. Materials and Methods

### 2.1. Materials

Ink was extracted from fresh squid (*Ommastrephes bartrami*) obtained from Zhou-Shan Fishery Company (Zhejiang, China) and stored at −40°C before use. For the preparation of samples with different metal contents, only nanopure water (>18.2 MX) obtained from a Simplicity TM system (Millipore, Billerica, MA) was used in the washing and for preparing solutions. The highest purity salts available (>99.99%, Sigma-Aldrich), including PbCl_2_, CdCl_2_, MgCl_2_, CaCl_2_, NaCl, EDTANa_2_, and NaOH, were used.

### 2.2. Preparation of Intact Squid Melanin

Melanin isolation and purification procedures have been described previously [22]. Briefly, ink was drained from the fresh ink sacs and diluted in nanopure water by at least 20-fold. The processes of centrifugation (5000 g, 15 min) and resuspension of the resulting pellet in water were repeated six times to remove water-soluble impurities present in the ink. The final pellet was resuspended in nanopure water and lyophilized to obtain the intact natural squid melanin. The sample, thus, obtained was examined by scanning electron microscope, which showed high purity without contamination by any cellular components [22].

### 2.3. Biosorption Studies 

#### 2.3.1. Effect of Metal Ion Concentration on Biosorption

Squid melanin's adsorption of Cd(II) and Pb(II) was tested by suspending 200 mg of the intact squid melanin powder in 50 mL of CdCl_2_ (99.999% pure, Aldrich) and PbCl_2_ (99.999% pure, Aldrich) solutions of varying concentrations at room temperature. Each mixture was centrifuged, and the upper solution was filtered and analyzed to determine the quantity of metal ions remaining.

#### 2.3.2. Influence of pH Value on Biosorption

For all the tests, 200 mg squid melanin was suspended in 2 mM/L metal ion solutions. Initially, pH was adjusted to values below the metal precipitation point to assure complete dissolution of each metal ion.

#### 2.3.3. Effect of Temperature on Biosorption

For all the tests, 200 mg squid melanin was suspended in 50 mL of 2 mM/L metal ion solutions. The temperature was raised incrementally from 15°C to 100°C for the analysis. The metal contents remaining in the solution were then analyzed.

### 2.4. Effect of Macrosalts on Biosorption

Varying concentrations of MgCl_2_, CaCl_2_, and NaCl were added to 50 mL of 2 mM/L heavy metal ion solutions, and the prepared solutions were each mixed with 200 mg of squid melanin. The salt concentrations varied from 1% to 5% for MgCl_2_ and CaCl_2_ and from 1% to 10% for NaCl. Subsequently, the metal contents remaining in the solution were analyzed.

### 2.5. Desorption of Heavy Metal-Enriched Squid Melanin

Tests were performed in much the same manner as the adsorption tests, except that in this case, they began with dried squid melanin that had previously been loaded with metal ions (using 2 mM/L solutions). The melanin was mixed with an eluent reactant solution at room temperature for 24 h and then centrifuged; the resulting solids were washed four times with nanopure water and analyzed for metal contents. Once the experiment was concluded, the squid melanin was collected for reuse in a new adsorption-desorption cycle. Two different reactants were used: HCl, at 0.5, 0.1, and 0.01 M/L concentrations, and EDTA, at 1, 10, and 40 mM concentrations. Each experiment was performed with 50 mL of solution.

### 2.6. Metal Content Analysis by Atomic Absorption Spectrophotometer

The metal ions in water were directly measured using an Atomic Absorption Spectrophotometer (AAS; Shimadzu AA 6800, Japan). Deuterium background correction was used, and the spectral slit width was 0.5 nm. The working currents/wavelengths for Pb(II) and Cd(II) were 10 mA/283.3 nm and 8 mA/228.8 nm, respectively. The instrument response was periodically checked with known metal solution standards. For each set of data presented, standard statistical methods were used to determine the mean values and standard deviations. Confidence intervals of 95% were calculated for each set of samples in order to determine the margin of error.

### 2.7. Amino Acid Analysis

The amino acid content of the melanin was measured by the method described previously [8]. Melanin (5 mg) was heated in 5 mL of 6 M HCl at 110°C for 24 h in an evacuated, sealed tube. The hydrolysate was evaporated under vacuum until dryness and was dissolved in 800 *μ*L of a pH 2.2 buffer for amino acid analysis. An aliquot of 0.1 mL was injected into the amino acid analyzer (Hitachi High Speed Amino Acid Analyzer L-8500, Hitachi Ltd., Tokyo, Japan).

### 2.8. Scanning Electron Microscopy (SEM) Assay of Squid Melanin

The squid melanin samples were coated with Au/Pd, and SEM was used to examine any morphological changes by means of an XL30 SEM-FEG scanning electron microscope (FEI Company, Hillsboro, Oregon, USA).

### 2.9. IR Analysis of Heavy Metal-Enriched Squid Melanin

All samples were freeze dried, and Fourier transform infrared spectra were recorded on a Bruker Vertex-70 FT-IR instrument equipped with a DTGS detector.

## 3. Results and Discussion

### 3.1. Biosorption 

#### 3.1.1. Effect of Metal Ion Concentration

**Figure 1:**
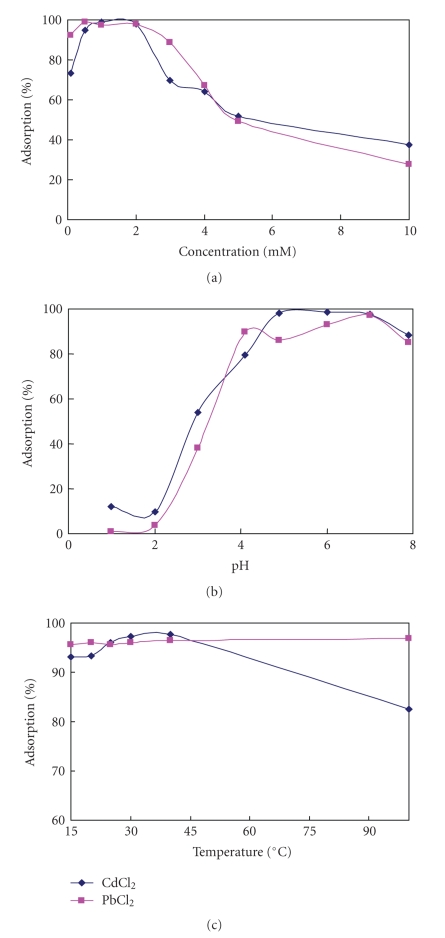
Absorption of heavy metals with squid melanin: (a) The influence of metal concentration on adsorption performance; (b) the influence of pH on biosorption performance; (c) the influence of temperature on biosorption performance.

The metal-removal efficiency (Figure 1(a)) indicates that for a concentration of 0.1 mM the removal rate was relatively low, accounting for 73% of the initial concentration of Cd(II) and 90% for Pb(II). However, at concentrations in the range of 0.5–2 mM, the uptake of both Cd(II) and Pb(II) was very high, accounting for 95% of the initial concentration. For higher metal concentrations, significant decreases in the Cd(II) uptake could be observed, which indicated that the adsorption capability was limited; however, Pb(II) uptake was still very high, about 90%, at a concentration of 3 mM/L. This might indicate that comparing to Cd(II), the adsorption of sepia melanin with Pb(II) was less affect by the concentration of metal, and they may have different groups in squid melanin accounting for the adsorption. Higher concentrations approached the saturation limit, with significant decreases in adsorption yields. The maximal adsorption rates of Cd(II) and Pb(II) by squid melanin were determined to be 105 mg/g and 135 mg/g, respectively, which are 0.93 mM/g of Cd(II) and 0.65 mM/g of Pb(II).

These results can be compared to the capacity of other biosorbents, both on a weight basis and in a batch system. The amount of Pb(II) that formed complexes with the squid ink (135 mg/g) was 2-fold higher than with either blast furnace sludge (64 mg/g) [24] or fungal mycelia byproducts (55 mg/g) [25]. For Cd(II), the complexation ratio (105 mg/g) was 3- to 7-fold higher than that for a rhamnolipid biosurfactant (45 mg/g) [26] or *Saccharomyces cerevisiae* (18 mg/g) [27]. Thus, the metal uptake capacity of squid ink melanin appears to be of great interest, given its superiority over other biosorbent efficiencies, as listed previously in a survey of biosorption of heavy metals by biomass materials [27].

#### 3.1.2. Effect of pH Value

The effect of pH on heavy metal uptake was investigated in the range of pH 1–8 at an initial ion concentration of 2 mM/L suspended with 200 mg of squid ink melanin (Figure 1(b)). Changes in the solution's pH were shown to have a significant affect on the uptake of Cd(II) and Pb(II). At pH levels below 3.0, both metals showed a poor uptake; adsorption yield increased significantly when the solution's pH value was changed from 3.0 to 4.0. Adsorption of both metals was very good in the range of 4.0–7.0 and then showed a reduction in uptake after 7.0. 

The low uptake of heavy metals at pH 3.0 may have been a result of hydrogen ions competing with copper ions to interact with the available binding sites. The reduced adsorption when the pH was greater than 7.0 might have been caused by neutralization of some of the carboxylic groups in the melanin by alkali, and the main adsorption site turned to be phenolic hydroxyl (OH) and amine groups (NH).

#### 3.1.3. Effect of Temperature

The uptake of both metals was not noticeably affected by changes in temperature in the range of 15–40°C (Figure 1(c)). Even when the temperature reached 100°C, the adsorption yield of Pb(II) was only slightly reduced. However, the adsorption of Cd(II) dropped significantly. This indicates that different adsorption or binding sites in squid melanin reacted with Cd(II) and with Pb(II), and this subject requires further investigation. Generally, squid melanin's adsorption of metals was not greatly affected by temperature, which suggests that cautions taken in previous research on the complexion of squid melanin with metal cations were unnecessary.

### 3.2. The Effect of Macrosalts

**Figure 2:**
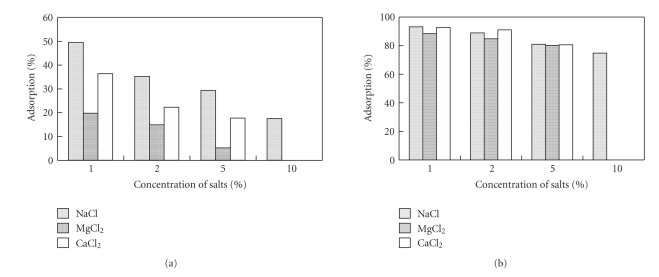
Effect of macrosalts on the biosorption performance: (a) Influence on the biosorption of Cd(II); (b) influence on the biosorption of Pb(II).

The effects of MgCl_2_, NaCl, and CaCl_2_, the salts that are most often used in natural products enriched with heavy metals, on the biosorption of Cd(II) and Pb(II) were studied (Figure 2). The salt concentrations ranged from 1% to 5% for MgCl_2_ and CaCl_2_ and from 1% to 10% for NaCl, as this compound appears in a relatively high concentration in some heavily salted foods. 

The presence of these salts was found to significantly affect the binding of Cd(II) (Figure 2(a)). When the concentrations of the three salts rose to 5%, the melanin's adsorption yield was only 30% for MgCl_2_, 19% for CaCl_2_, and 7% for NaCl. This indicates that the Cd(II) binding was interfere with these salts. In other words, Cd(II) competed with Na(I), Ca(II), and Mg(II) for COOH group in squid ink melanin; thus, squid melanin can only be used to remove Cd(II) from solutions with a low concentration of salts.

However, all three salts had a minor effect on adsorption of Pb(II) (Figure 2(b)). Even when the concentration of NaCl was very high, 10% of the solution, the Pb(II) adsorption rate was 74%. Likewise, the addition of various concentrations of MgCl_2_ and CaCl_2_, from 1% to 5%, scarcely affected the adsorption of Pb(II); the yield was reduced slightly but still exceeded 80%. This is unequivocal evidence that Na(I), Ca(II), and Mg(II) bind to the COOH group, and Pb(II) mainly biding to the other sites. Furthermore, the salts binding does not interfere with Pb(II) binding. Squid melanin could successfully be used to remove Pb(II) from products containing these salts.

### 3.3. Desorption

Regeneration of a biosorbent for repeated use is a critical issue in practical application. The recovery of heavy metals from metal-laden biomass has been approached by utilizing various desorption agents, including HCl, H_2_SO4, Na_2_CO_3_, EDTA, and mercaptoethanol [28–31]. Among these approaches, decreasing the pH value using HCl and EDTA appears to have had the best desorption efficiency and was thus selected as the desorption agent in the present study. To determine the optimal HCl and EDTA concentrations for metal desorption, the amount of metals released from treated squid melanin at different concentrations of desorption agents was observed in Table 1. Briefly, it appears that both Cd(II) and Pb(II) can be almost completely recovered by washing twice with 0.01 M HCl, about 95% and 98.5%. An increase in the acid or EDTA concentration resulted in a more complete desorption of Cd(II) and Pb(II); the desorption rate can exceed 99.5%. After desorption, the squid melanin was reapplied to 2 mM/L metal ion solutions, with adsorption rates of 88.6% and 92.3%, respectively; this indicates that squid melanin is a reasonable natural source to absorb heavy metal contaminants.

### 3.4. IR Analysis

Binding of metal ions was expected to affect the transition frequencies of the coordinated functional groups. If metal ions bound to a COO–group, then concentration-dependent changes would appear in the infrared region characteristic of the C=O stretching frequency of the acid moiety. Likewise, if ions are bound with OH or NH groups, we would anticipate changes in the infrared spectrum (loss of N-H and O-H intensity) reflecting deprotonation to accommodate binding of metal cations. Previously reports about analyses of the infrared spectra of natural and synthetic melanins indicate the following. In indole or pyrrole systems, For NH (~3200 cm^−1^) and OH stretching (~3400 cm^−1^), the peak assignments were 3200–3500 cm^−1^. For COOH and C=O stretching, the peak assignment was 1710 cm^−1^; for aromatic C=C and C=N bending and C=O stretching (non-carboxylic acid), it was 1590–1690 cm^−1^; for C=O stretching ionized COO–, it was 1580 cm^−1^; and for phenol OH and the carboxyl OH, it was 1250 cm^−1^.

**Figure 3:**
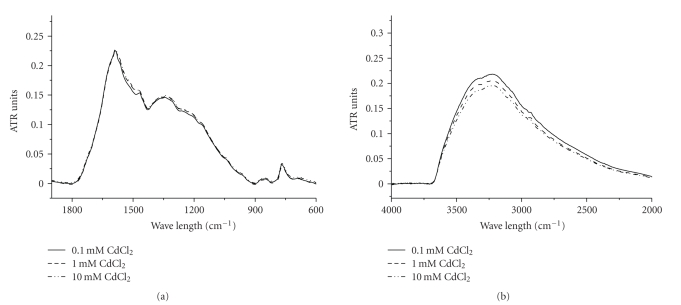
IR spectrum of heavy metal enriched squid melanin as a function of the solution concentration of Cd(II) in spectral region of (a) 900–1900 cm^−1^; (b) 2000–4000 cm^−1^. The spectra are normalized to the intensity of the 1598 cm^−1^ peak.

**Figure 4:**
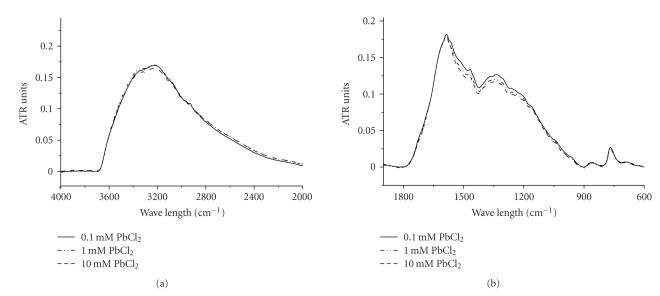
IR spectrum of heavy metal enriched squid melanin as a function of the solution concentration of Pb(II) in spectral region of (a) 900–1900 cm^−1^; (b) 2000–4000 cm^−1^. The spectra are normalized to the intensity of the 1598 cm^−1^ peak.

All the tests on this subject were conducted in pH 6.0 conditions. Figures 3 and 4 show the effect of added metal on the intensity of observed infrared transitions. With increasing concentrations of bound Cd(II), the intensity of the protonated NH (3200 cm^−1^) and phenolic OH (3400 cm^−1^) bands decreased notably (Figure 3(b)), which indicated deprotonation of the OH group of phenolic groups upon metal binding. However, the intensity of the COOH (1710 cm^−1^) adsorption band was not affected (Figure 3(a)).

In contrast to Cd(II), the intensity of the COOH adsorption band increases upon the binding of Pb(II) for solution concentrations increase (Figure 4(a)). At the same time, the intensity of the OH and NH band decreases (Figure 4(b)). This suggests that unlike Cd(II) binds only to the catechol groups and amino groups, Pb(II) also binds to the carboxyl group.

All these results indicate that phenolic hydroxyl (OH), carboxyl (COOH), and amine groups (NH) are likely potential binding functional groups for metal ions. However, the specific binding sites for Cd(II) and Pb(II) are different.

### 3.5. SEM Assay

**Figure 5:**
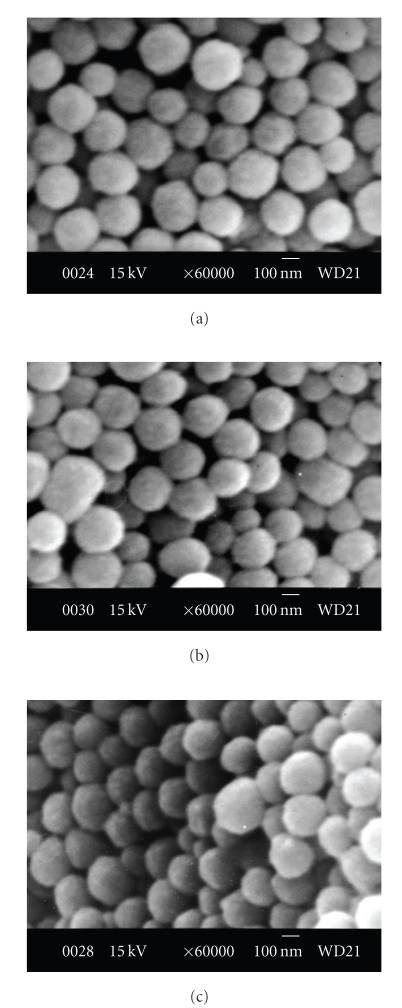
SEM images of squid melanin with heavy metals. (a) Natural squid melanin; (b) Cd(II)-enriched (105 mg/g); (c) Pb(II)-saturated (135 mg/g). The scale bar in each panel corresponds to 100 nm.

Resuspended squid melanin was exposed to a 5 mM solution of Cd(II) (or Pb(II)), and the resulting preparations were washed and freeze-dried. Amino acid analysis indicated no change of amino acid composition. SEM images (Figures 5(a), 5(b) and 5(c)) of the squid melanin granules before (Figure 5(a)) and after binding with Cd(II) (Figures 5(a) and 5(b)) and Pb(II) (Figure 5(c)) confirm that binding to iron does not affect their morphology; the granules are nearly spherical in shape, with a mean diameter of 50 ~ 150 nm. Further DSC curve analysis indicated that the squid melanin's stability rises after it is enriched with heavy metals (data not shown).

## 4. Conclusion

The biosorption of two heavy metals, Cd(II) and Pb(II), using squid melanin was evaluated in the present study. At a concentration of 2 mM/L metal ions, 95% adsorption efficiency was reached for both Cd(II) and Pb(II), with maximum binding contents of 0.93 mM/g and 0.65 mM/g, respectively. Temperature had no obvious effect on the adsorption of the metals, and in a pH range from 4.0 to 7.0, the adsorption yield was high and stable. Salts had no obvious effect on the binding of Pb(II), though they greatly reduced the adsorption of Cd(II), suggesting that they bound to the same functional groups in squid melanin. Infrared analysis revealed phenolic hydroxyl (OH), carboxyl (COOH), and amine groups (NH) as the possible functional groups responsible for metal binding in squid melanin; however, the specific binding sites for Pb(II) and Cd(II) were different. Squid ink is the waste of squid products processing; it has caused a lot money and labor to disposal. However, there was no report on reasonable utilize of it, especially melanin, which is the main component of squid ink. We have thus demonstrated squid melanin's potential as a new material for removing heavy metals from low-strength wastewater, an application not previously considered by other scientists.

## Figures and Tables

**Table 1:** Effect of different concentration of EDTA and HCl on desorption of Pb(II).

Desorption agents	Desorption rate (%)	
	Cd(II)	Pb (II)
0.5 M HCl	99.82	99.88
0.1 M HCl	99.46	99.83
0.01 M HCl	98.62	99.48
1 mM EDTA	95.06	98.59
10 mM EDTA	98.54	99.79
40 mM EDTA	99.76	99.83
